# Where Can Artificial Intelligence Assist Cancer Care?: Examining Patient‐Centered Communication Dimension Effects

**DOI:** 10.1111/1475-6773.14653

**Published:** 2025-06-06

**Authors:** Qiwei Luna Wu, Yue Liao, Grace Ellen Brannon

**Affiliations:** ^1^ Department of Communication Studies College of Media & Communication, Texas Tech University Lubbock Texas USA; ^2^ Public Health Programs, Department of Kinesiology College of Nursing and Health Innovation, University of Texas Arlington Arlington Texas USA; ^3^ Department of Communication College of Liberal Arts, University of Texas Arlington Arlington Texas USA

**Keywords:** artificial intelligence, cancer care, patient‐centered communication, structural equation modeling, telehealth

## Abstract

**Objective:**

To explore how aspects of patient‐centered communication (PCC) may directly or indirectly predict patients' preferences for artificial intelligences (AIs) versus human medical professionals, based on the stimulus‐organism‐response model.

**Study Setting and Design:**

As AI gains popularity and researchers explore its application in the medical context, it is important to understand how current patient‐provider dynamics involving high technology (e.g., telehealth communication) may shape patients' perceptions of future use of AI, especially in the context of cancer care where patient satisfaction and sense of care continuity are important. Participants were recruited from an online panel in China (June 2024). Structural equation modeling analyzed the relationships among variables, including six PCC dimensions (i.e., exchanging information, fostering healing relationships, making decisions, managing uncertainty, responding to emotions, and enabling patient self‐management), communication outcomes (i.e., patient satisfaction, sense of care continuity), and patients' preference of AIs vs. human medical professionals.

**Data Sources and Analytic Sample:**

Primary data were collected from an online panel of 495 Chinese cancer patients in China, representative of the gender and age distribution of the overall Chinese population due to quota sampling.

**Principal Findings:**

Direct predictors of preference for replacing human medical professionals with AIs included lower patient satisfaction (*β* = −11, *p* < 0.05), lower ease of use (*β* = −0.1, *p* < 0.05), better care continuity (*β* = 0.15, *p* < 0.01), providers' attending to emotions (*β* = 0.17, *p* < 0.05), and less enablement in self‐management (*β* = −0.17, *p* < 0.01). Patient satisfaction, ease of use, and care continuity mediated the relationships between different PCC dimensions and patients' preferences for AI use.

**Conclusions:**

PCC and communication outcomes are associated with cancer patients' preferences in future AI use. Our study sheds light on how clinicians may improve their communication to educate patients on navigating the cancer care continuum using AI technology.

**Summary**

- What is known on this topic
  ○ Artificial intelligence is gaining popularity, yet its application in the medical context, specifically in the oncology context, is unknown.
  ○ Patient‐centered care is especially important in the context of cancer care where patient satisfaction and sense of care continuity are vital to improving health outcomes.
  ○ There is some concern of artificial intelligence replacing human medical providers, yet little research examines how artificial intelligence may complement human care.
- What this study adds
  ○ Patient‐centered communication, specifically attention to emotions, is associated with preferences for using artificial intelligence in healthcare in the future.
  ○ Patients having lower satisfaction with healthcare providers had higher intentions to use artificial intelligence in more treatment methods.
  ○ Our findings did not show that patients believe that artificial intelligence *replaces* human provider communication; rather, there is a possibility for artificial intelligence to *supplement* poor provider communication.

## 1 Introduction

Artificial intelligence (AI, defined as advanced computation methods allowing machines to mimic human cognitions functions like learning or problem‐solving) is increasing in popularity, particularly in the healthcare context [1]. These can include large language models (e.g., OpenAI's ChatGPT). While it can help with diagnosis, treatment planning, drug discovery, and monitoring in the oncological context [2], researchers discuss AI's potential to replace human healthcare providers, with some suggesting human providers work alongside AI [3, 4]. Nevertheless, providers may perceive logistical constraints such as AI's compatibility with existing systems, potentially causing delays in clinical adoption; patients also reported concerns about AI, including data quality and privacy [3]. Other potential harms include algorithmic bias and inadequate or missing informed consent processes [5]. Replacement or not, much is unknown about how AI can optimize healthcare services when humans fall short. Therefore, it is important to understand how current patient‐provider dynamics involving high technology (e.g., telehealth communication) may shape patients' perceptions of future use of AI, especially in the context of cancer care where patient satisfaction and sense of care continuity play essential roles. Telehealth was specifically chosen as the context for the present study given that people choose telehealth for its convenience and accessibility, as it allows them to receive care from home, and these unique aspects—like reduced travel time and flexible scheduling—can significantly shape patient preferences toward more timely and comfortable healthcare experiences [6, 7].

In this study, we explore potential sources of patients' AI preferences over human healthcare providers by examining the relationship among providers' patient‐centered communication, patient outcomes, and patient perceptions of AI superiority. Our study will contribute to the understanding of patients' technological preferences, as well as where AIs can efficiently supplement human interactions.

### 1.1 Clinical Environment and Where It Falls Short

Patient‐centered communication (PCC) is defined as interactions between patients and healthcare providers so as to meet patients' preferences and needs [8]. PCC components include exchanging information, fostering healing relationships, making decisions, managing uncertainty, responding to emotions, and enabling patient self‐management, which in turn can affect communication and ultimately health outcomes [9, 10, 11]. In the cancer context, providers' responsiveness to patient uncertainty and negative emotions predicted better mental health, physical health, coping, and less psychological distress during the COVID‐19 pandemic [12]. PCC, as a gold standard of quality health care, is also predictive of other patient outcomes, such as patient satisfaction and sense of care continuity [6].

Yet, research documented that the prevalence of optimal PCC decreased during COVID‐19 (e.g., 2020) in all domains except for exchanging information [13]. With the shortages of healthcare supplies during a health crisis like COVID‐19, it is perceivable that human healthcare providers had their limits.

Similarly, organizational factors within the clinical environment, such as wait time, are closely related to patients' evaluations of their care [14, 15]. Extended wait times before a consultation are commonly associated with lower patient satisfaction [14, 15]. As demand for healthcare services continues to rise in Chinese hospitals, the adoption of telehealth has helped reduce wait times in many regions [7, 16].

Indeed, where the clinical environment falls short, technology can fill in. Patients reported better perceived PCC when they frequently used online patient portals to navigate health records and communication with providers outside of the clinics [17]. However, as AI gains popularity, how this new technology can enhance healthcare experiences is underexplored. Using the stimulus‐organism‐response (SOR) model, we investigate AI's potential to supplement specific aspects of PCC.

#### 1.1.1 Stimulus‐Organism‐Response Theory as a Framework

SOR provides a theoretical framework for interpreting user behavior [18, 19, 20]. Within SOR, the stimulus (S) is a specific external or environmental factor affecting the individual‐level psychological or perceptual state [21]. It is a ubiquitous indicator marker in the physical environment [22]. The stimuli can trigger the cognitive and emotional organism (O) within the user, leading to the behavioral response (R) of the user being triggered [19].

SOR is frequently used to study user adoption of mobile apps [23, 24] and further the relationship between external stimuli on consumer sentiment in mobile social commerce and their relationship with consumer behavior [25]. Research also used this model to study how PPC and health‐related social media use affect cancer survivors' meaning in life [26, 27]. Other research showed how human‐to‐system interaction (e.g., how easily people believe they can interact with the functions of a specific platform) has a stronger impact on the outcome expectation of health self‐management competence than that of human‐to‐human interaction [26, 28].

In the cancer care context, patients may react to declining patient‐centered communication they experienced (S) and extended wait time (S) with adverse reactions, such as low patient satisfaction and diminished sense of care continuity (O). As a result, they may turn to alternative information sources and care options (R), including online health‐related information and AI technologies, to compensate for the possible lack of support in their health care [29]. The literature has long cautioned patients about the proliferation of cancer‐related misinformation and harmful information in online information‐seeking [30]. Research on patients' use of AI is relatively new, primarily focusing on patients' preferences and perceived challenges (e.g., data‐source bias, misinformation, fake testimonies) [31, 32]. However, much like how patients' information‐seeking behavior can sometimes facilitate productive conversations with their healthcare providers, the use of AI (especially when approached with patient‐centeredness) may become powerful in supplementing the limitations of human services (e.g., non‐instant reporting) [30, 33, 34].

### 1.2 AI Superiority and Intentions to Adopt

Despite heated debate over AI's replacement of human tasks [35, 36], the literature acknowledged the potential of the AI technology in speedy processing of diverse data types (e.g., laboratory findings, images, and doctors' notes), diagnostic accuracy, and improved capabilities in showing empathy, all of which are essential in patients' perceptions of care quality [37, 38, 39]. One recent retrospective case‐cohort study found that when using a negative screening examination, for example, AI algorithms performed better than a manual risk model (the breast cancer surveillance consortium risk model) for predicting breast cancer risk at 0–5 years after the initial mammographic examination; combining both models further improved predictions [40].

**FIGURE 1:**
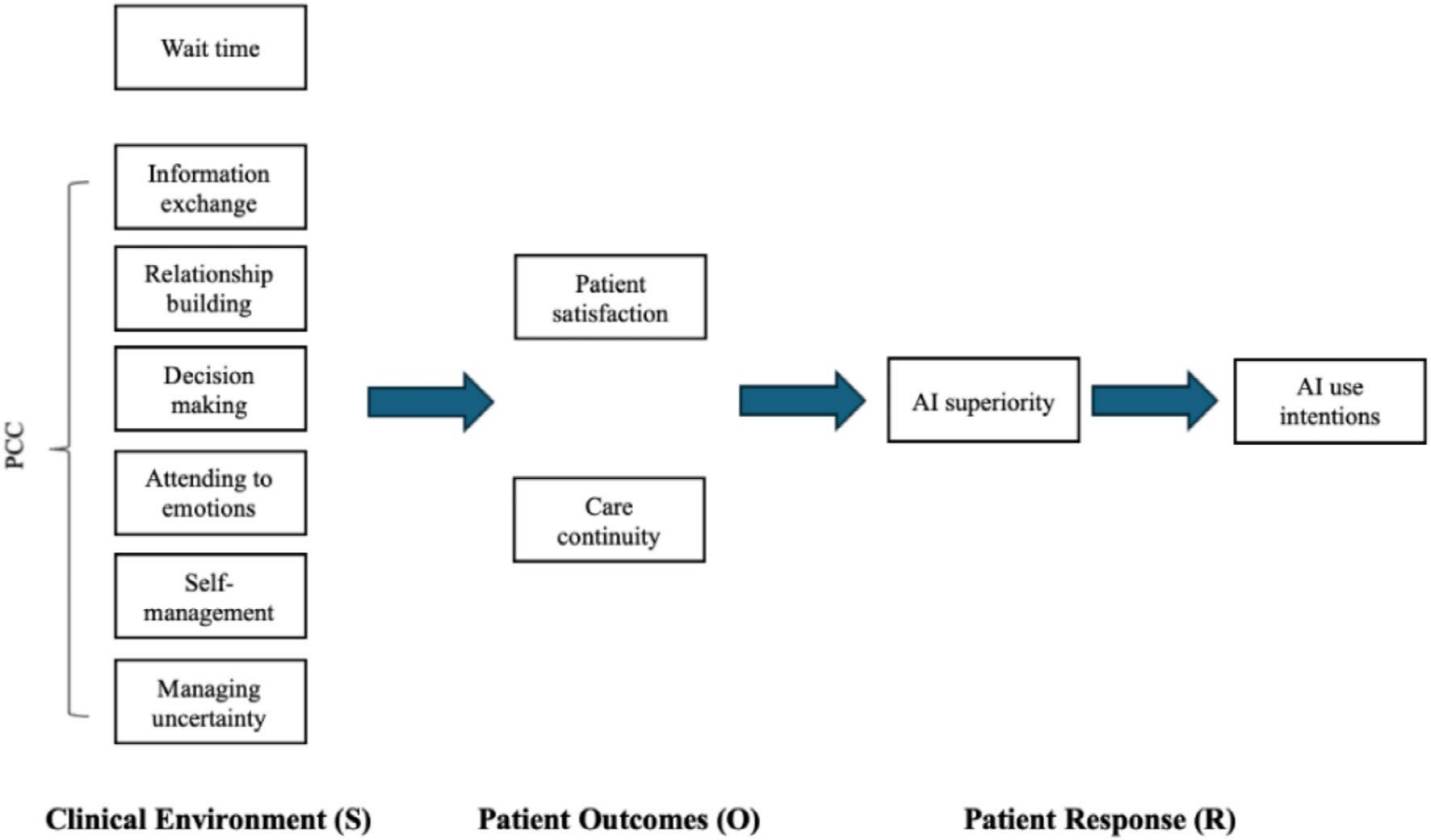
Theoretical model describing relationships between variables and theory. Abbreviations: AI, artificial intelligence; O, organism; PCC, patient‐centered communication; *R*, response; S, stimulus.

Drawing on the SOR theory, limitations of human‐run clinics (S)—long wait time, inefficient exchange of information due to patient‐provider power difference, inability to address patients' emotions, and challenges to build patient rapport in interracial contexts—may result in patients' frustration (e.g., low satisfaction, low sense of care continuity; O), leading to compensational beliefs in AI superiority (R) [6, 41, 42]. Hence, we hypothesize (Figure 1):

*Negative organizational (*e.g., *wait time) and communicative aspects (*e.g., *PCC dimensions) of patient care may be related to patients' belief in AI superiority over human healthcare professionals*.

Along the same line, as attitudes and beliefs are precursors to behavioral intentions [43], patients who think highly of AI's ability may be motivated to use AI‐powered automatic medical examinations, such as diagnosis, medication prescriptions, and interpretation of lab results [44]. We hypothesize:

*Negative features of organizational (*e.g., *wait time) and communicative aspects (*e.g., *PCC dimensions) of patient care may be related to patients' preference for AI‐powered healthcare services to those conducted by human healthcare professionals*.

## 2 Methods

### 2.1 Study Design

The observational study was conducted online and follows the STROBE reporting guidelines [45]. The protocol IRB‐FY2024‐254 was approved by the Institutional Review Board at INSTITUTION REDACTED on June 14, 2024.

### 2.2 Participants and Sampling

In July 2024, we conducted this study through a Chinese crowdsourcing company that conducts questionnaire research, Wenjuan (https://www.wenjuan.com/). Wenjuan recruited adult Chinese cancer patients based on the inclusion criteria of (1) who used telehealth services in the past year, and (2) would like to use telehealth in the future (*n* = 495). This sample roughly represents the age and gender distribution of the Chinese cancer population due to quota sampling.

### 2.3 Measures

The six dimensions of PCC were measured by a 36‐item PCC scale [46]. Across the subscales of exchanging information (six items), fostering healing relationships (seven items), making decisions (five items), responding to emotions (six items), enabling patient self‐management (six items), and managing uncertainty (six items), different five‐point response option formats were used to assess different aspects of PCC. These options included frequency (1 = *never*, 5 = *always*), amount (1 = *not at all*, 5 = *a great deal*), and quality (1 = *poorly*, 5 = *outstanding*). A higher point value represents a better PCC evaluation. For detailed item information, see Supporting Information.

*Wait time* was measured by one item from previous literature, “For your most recent telehealth visit, how long did you wait online before your provider joined the telehealth session?” [6]. Three response options to this question included “did not wait—provider joined immediately (1),” “waited 5–10 min (2),” and “waited longer than 10 min (3).” The mean wait time on this scale was 2.19 (SD = 0.80), indicating the average time was a little over the “5–10 min wait.” [14, 16].

Patient outcomes of telehealth visits included *patient satisfaction* and sense of care *continuity* were each measured by an item from previous literature [6]. Answers were measured on a five‐point scale (1 = *strongly disagree*, 5 = *strongly agree*). The item for *satisfaction* was “I was very satisfied with the care I received during that visit” (M = 4.15, SD = 0.63). For sense of care *continuity*, the item reads “Receiving care via a telehealth visit provided me with a sense of access and continuity of care with my provider/provider's practice during the pandemic” (M = 4.39, SD = 0.56).

Patients' preferential belief about AIs versus human medical professionals (HMPs) was measured by one item synthesized from the previous literature [47], “How would you rate your preference of a human medical professional vs an AI?” Answers ranged from “Strongly prefer human medical professional (1),” “Slightly prefer human medical professional (2),” “Neutral (3),” “Slightly prefer AI (4),” to “Strongly prefer AI (5)” (M = 2.17, SD = 1.47).

The primary outcome, patients' *intention* in allowing AI to participate in treatment, was measured by the sum of six dummy items synthesized from the previous literature [47]. The dummy items asked patients if they preferred a human medical professional (0) or an AI (1) in situations including (a) prescribing pain medications, (b) deciding when to go to the emergency room, (c) diagnosing a rash in a sensitive area, (d) diagnosing a rash on your arm, (e) reading a scan (X‐ray, mammogram, MRI), and (f) managing your diet. The sum score of the items ranged from “0” (totally preferred a human medical professional) to “6” (totally preferred an AI).

Covariates included age (18 ~ 30/31 ~ 40/41 ~ 50/51 ~ 64/65+), gender (man/woman), education (less than high school/some high school/high school or equivalent (e.g., GED)/some college, including associate degree or trade school/bachelor's degree or higher/master's degree or higher/doctorate or professional degree), and cancer stage (stage 0/1/2/3/4/5).

The questionnaire was originally created in English but then translated to Chinese using google translate (translate.google.com). One of the researchers who speaks both Chinese and English polished the google translation to correct grammar mistakes for a more accurate translation. This was then backtranslated from Chinese to English using google translate again to verify the validity of the language. After identifying high similarity between the original questionnaire and the backtranslation, we finalized the questionnaire in Chinese.

### 2.4 Analysis

We used bivariate analysis to explore the correlations between the predictors and outcome without covariates to determine the unadjusted effects. We used structural equation modeling (SEM) to test the hypotheses. We tested a just‐identified model controlled for age, gender, education, and cancer stage, with a non‐directional path linking patient satisfaction and sense of continuity to account for shared variance of both patient outcome aspects [6]. The maximum likelihood method was used to estimate path parameters. Kline's [48] cutoff indices were used to test the model's goodness‐of‐fit (i.e., an insignificant chi‐square test, or RMSEA < 0.08, CFI ≥ 0.90, and SRMR < 0.08). Standardized path coefficients were reported for the SEM model.

## 3 Results

### 3.1 Descriptive Analysis

Of the 495 participants, 280 were male (56.6%) and 215 were female (43.4%). Most participants were in the age group of 41–50 (*n* = 270, 54.5%). About 67.7% of the participants (*n* = 335) finished high school or above. A relatively normal distribution was observed in the cancer stages, as 36.6% of the participants reported Stage II diagnosis (*n* = 181), followed by 33.1% of the participants (*n* = 164) reporting Stage I cancer (Table 1).

**TABLE 1:** Demographics of participants and variable descriptives.

	*N*	%	M	SD
Age				
18–30	7	1.4		
31–40	59	11.9		
41–50	270	54.5		
51–64	153	30.9		
65+	6	1.2		
Gender				
Male	280	56.6		
Female	215	43.4		
Education				
Less than high school	66	13.3		
Some high school	94	19		
High school or equivalent (e.g., GED)	181	36.6		
Bachelor's degree or higher	142	28.7		
Master's degree or higher	12	2.4		
Cancer Stage				
Stage 0	20	4		
Stage I	164	33.1		
Stage II	181	36.6		
Stage III	122	24.6		
Stage IV	8	1.6		
Preference			2.17	1.47
PCC_information			4.07	0.454
PCC_relationship			4.2	0.393
PCC_decision			4.06	0.707
PCC_emotions			4.17	0.416
PCC_SelfCare			3.89	0.811
PCC_uncertainty			4.01	0.504
Wait time			2.19	0.795
Satisfaction			4.15	0.633
Continuity			4.39	0.557

Abbreviation: PCC, patient‐centered communication.

### 3.2 Bivariate Analysis

Bivariate analysis showed that patients' increased intention to use AI in their treatment was correlated with better perceived treatment continuity (*r* = 0.16, *p* < 0.01) and higher preference of AI to human medical professionals (*r* = 0.49, *p* < 0.01) (Table 2).

**TABLE 2:** Bivariate analysis of key study variables.

	(1)	(2)	(3)	(4)	(5)	(6)	(7)	(8)	(9)	(10)	(11)
(1) Intention	1										
(2) PCC_information	−0.04	1									
(3) PCC_relationship	0.04	0.66***	1								
(4) PCC_decision	0.05	0.35***	0.41***	1							
(5) PCC_emotions	0.04	0.63***	0.73***	0.46***	1						
(6) PCC_self management	−0.08	0.24***	0.30***	0.35***	0.34***	1					
(7) PCC_uncertainty	0.004	0.50***	0.54***	0.35***	0.61***	0.47***	1				
(8) Wait time	0.06	−0.08	−0.08	−0.06	−0.03	−0.05	−0.02	1			
(9) Satisfaction	−0.02	0.22***	0.28***	0.18***	0.28***	0.18***	0.20***	−0.04	1		
(10) Continuity	0.16***	0.24***	0.35***	0.15***	0.32***	0.12***	0.22***	−0.10**	0.24***	1	
(11) Human versus AI	0.49***	−0.05	−0.06	−0.02	0.02	−0.10**	−0.02	0.10**	−0.11**	0.08	1

*Note:* **p < 0.05, ***p < 0.01.

Abbreviations: AI, artificial intelligence; PCC, patient‐centered communication.

### 3.3 SEM Analysis

**FIGURE 2:**
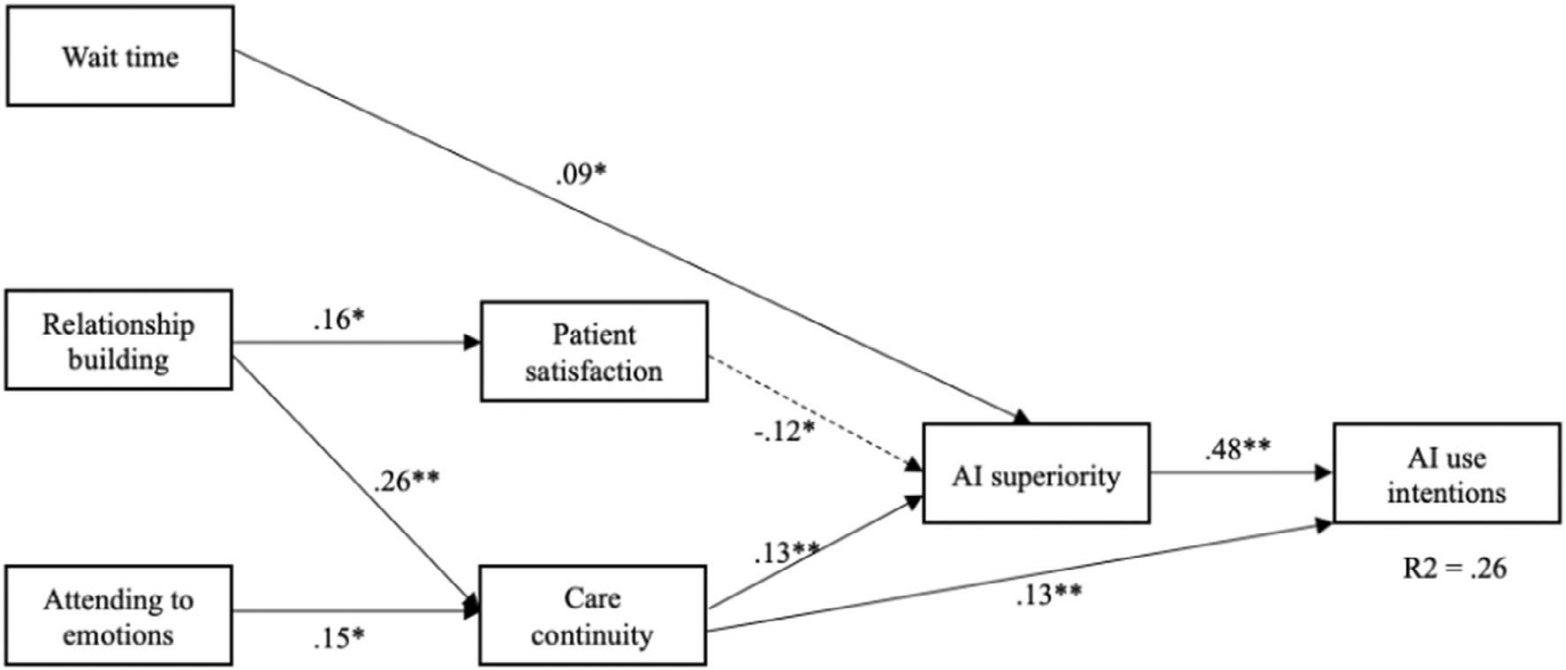
SEM model showing predictors of AI use intentions. *Note*: *p < 0.05; **p < 0.01; AI = artificial intelligence.

The original model generated a perfect fit due to its being just‐identified (χ^2^_(0)_ = 0, *p* < 0.01, RMSEA < 0.01, SRMR < 0.01, CFI = 1) (see Appendix S1 for details). To strengthen the interpretation of the model [49], we tested a new model without the insignificant paths from the five PCC dimensions and patients' intentions. It showed that the new model fit the data well (χ^2^_(5)_ = 7.1, *p* = 21, RMSEA = 0.03, SRMR = 0.01, CFI = 0.99). It explained 26.2% of the variance in the primary outcome, patients' intentions to use AI in their treatments. This model was accepted as the final model (see Figure 2, Table 3).

**TABLE 3:** Pathways linking patient satisfaction, care continuity, AI superiority, and AI preference: Structural equation modeling results.

	Patient satisfaction		Care continuity		AI superiority		AI preference	
	*ß*	*p*	*ß*	*p*	*ß*	*p*	*ß*	*p*
Age	−0.03	0.5	−0.02	0.65	0.04	0.34	0.01	0.88
Gender	−0.03	0.42	−0.01	0.75	−0.02	0.59	−0.02	0.6
Education	−0.03	0.49	0.02	0.59	0.06	0.2	0.01	0.85
Cancer stage	0.003	0.94	−0.01	0.86	0.06	0.17	−0.03	0.44
Wait time	−0.02	0.69	−0.08	0.06	0.09	0.04	0.02	0.53
PCC information	0.02	0.74	−0.03	0.61	−0.05	0.4	−0.05	0.21
PCC rapport	0.16	0.02	0.26	< 0.01	−0.1	0.16		
PCC SDM	0.03	0.56	−0.03	0.58	−0.02	0.77		
PCC emotion	0.12	0.08	0.15	0.03	0.13	0.07		
PCC self‐management	0.09	0.07	0.01	0.91	−0.1	0.06		
PCC uncertainty	−0.03	0.64	−0.003	0.96	0.01	0.84		
Patient satisfaction					−0.12	0.01	0.02	0.59
Care continuity					0.13	0.01	0.13	< 0.01
AI superiority							0.48	< 0.01
*R* ^2^	0.1		0.14		0.06		0.26	

Abbreviations: PCC, patient‐centered communication; SDM, shared decision‐making.

As a result, patients' preference of using AI over HMP (*β* = 0.48, *p* < 0.01) and better sense of treatment continuity (*β* = 0.13, *p* < 0.01) significantly predicted patients' intentions to use AI in more treatment methods.

Providers' better ability to build rapport (*β* = 0.26, *p* < 0.01) and address patients' emotions (*β* = 0.15, *p* < 0.05) was significantly associated with a better sense of care continuity.

Lower patient satisfaction (*β* = −0.12, *p* < 0.05), better care continuity (*β* = 0.13, *p* < 0.01), and longer wait time (*β* = 0.09, *p* < 0.05) were significantly associated with patients' perceptions of AI superiority.

Providers' better ability to build rapport (*β* = 0.16, *p* < 0.05) was significantly associated with patient satisfaction.

## 4 Discussion

Using a sample of cancer patients residing in China, this study found that higher intention to use AI was associated with better perceived treatment continuity and longer wait time, but lower patient satisfaction. While most literature examined the scheduling wait time (i.e., time between telehealth appointment scheduling and the actual appointment) for Chinese cancer patients (being between less than 24 h) [16], we assessed the wait time in the digital waiting room. Compared to the roughly 40 min wait in an out‐patient Chinese hospital, the digital wait time (a little more than 5–10 min) is significantly lower [14]. Nevertheless, abundant literature showed that long wait time is one of the most significant predictors of patient dissatisfaction in a variety of healthcare settings [15], including those in China [14]. As wait times in the cancer context have increased over the past decade [50], our findings imply that patients may be more open to using AI in efforts to use their time more efficiently. This finding may demonstrate that satisfaction may be less important than getting seen and initiating treatment, particularly for cancer patients, who may have a variety of upcoming appointments including imaging referral, biopsies, biomarker reports, medical oncology consultation, radiation oncology consultations, and surgical consultations, and that delays in each of these areas add up substantially.

One key finding is that perceiving AI as superior to HMP predicts future AI use intentions. Feeling that AI is accurate, and perhaps makes fewer mistakes than humans, may in fact help patients achieve their wanted health outcomes. Previous studies that examined AI use in patients facing end‐of‐life decisions found that using AI and wearable devices successfully predicted patient death within 7 days, which supports clinical decision‐making practices for personalized patient care [51]. This resonates with the literature on the superior diagnostic accuracy of AI [37], especially when combined with human analysis. With the flexibility, high‐speed big data processing, and scalability of AI applications in health care [37], it is possible that AI may be a strong factor in accurate decision making in the future.

It is somewhat surprising that PCC variables were not more significantly associated with patient satisfaction and care continuity, yet other studies have shown that PCC may not always predict patient satisfaction due to social and cultural reasons (e.g., collectivism and respect for authorities like healthcare providers). Satisfaction may be evaluated differently in China, based on not only interpersonal skills but also ethics (e.g., not taking bribes) [52]. While previous literature reported the decline of optimal PCC experienced by cancer patients after the COVID‐19 pandemic [13], it is important to recognize that AI may have the potential to supplement poor provider communication. Even so, our findings did not show that patients believe that AI *replaces* human provider communication. This is in line with researchers' recommendations that humans and AI can work better together than alone [37, 53], and AI may not completely replace human healthcare services especially when it comes to empathy and interaction quality [54].

Other research has shown that patient perceptions associated with future use of telehealth included higher care continuity, and that PCC does not directly predict future use of telehealth [6]. Our research therefore complements and extends this previous finding by focusing on AI specifically, with our findings also demonstrating that PCC does not directly predict future use of AI. Yet, our findings also differ from the same study. While they found that patient perceptions associated with future use of telehealth were associated with higher patient satisfaction, our findings were that patients' intentions to use AI in more treatment methods were significantly associated with lower patient satisfaction. It is possible that this can be explained by the potential mediator of superiority perceptions. Specifically, there may be a difference between telehealth and AI in that there may be cognitive differences between the framing of AI superiority (e.g., a preference for AI in general) instead of a preference for using AI with specific intention (e.g., for specific tests of concerns).

### 4.1 Implications

While the US national surveys showed that American cancer patients' optimal PCC experiences significantly declined after the COVID‐19 pandemic [13], our findings are especially relevant for United States cancer care. Given the diverse immigrant populations, AI (e.g., ChatGPT, Gemini) could help American patients with limited English proficiency seek the quality care they deserve. For instance, AI translation services can particularly be helpful for healthcare providers to support marginalized populations (e.g., refugees). This use of AI has the potential to alleviate pressure on family members who may not be qualified to be a medical translators, particularly for children who may not even know what they are translating and perhaps may be exposed to information their parents or guardians do not prefer them to have but instead must disclose out of necessity.

Future AI implementation should also consider informed consent processes and privacy of data storage to reduce concerns of privacy violations. Concerns about how AI and healthcare organizations alike may exist. Healthcare organizations should develop clear processes and standard operating procedures for detailing consenting processes and patient notification pathways for informing patients, families, and caregivers for transparent communication to alleviate potential concerns.

### 4.2 Limitations

While AI itself is defined in research, it is possible that survey participants chose to operationalize AI how they saw fit. Future research examining uses of AI in the healthcare context may choose to provide examples to ensure all participants, particularly those less familiar with AI, have a similar understanding of AI. Further, the research presented in the manuscript is cross‐sectional in design; future research should examine these processes longitudinally.

### 4.3 Conclusion

Given that virtual healthcare delivery is increasing since COVID‐19, with research showing potential avenues of integrating AI into healthcare delivery, there is great opportunity for AI to supplement traditional methods of healthcare delivery. It is important to identify strategies to ethically implement AI in healthcare delivery to improve patient experience and health outcomes alike.

## Author Contributions

**Qiwei Luna Wu:** conceptualization, data curation, formal analysis, investigation, methodology, project administration, resources, software, supervision, validation, visualization, writing – original draft, writing – review and editing. **Yue Liao:** investigation, visualization, writing – original draft, writing – review and editing. **Grace Ellen Brannon:** conceptualization, data curation, investigation, methodology, project administration, resources, supervision, validation, visualization, writing – original draft, writing – review and editing.

## Conflicts of Interest

The authors declare no conflicts of interest.

## Supporting information


**Data S1.** Items from the PCC‐CA‐36 measure arranged by subdomain with relevant statistical information.

**Appendix S1.** Supporting Information.
