# Supplementary material for: Where Can Artificial Intelligence Assist Cancer Care?: Examining Patient‐Centered Communication Dimension Effects
Source: Health Serv Res. 2025 Jun 6;61(2):e14653. doi: 10.1111/1475-6773.14653 (PMC12967909; doi:10.1111/1475-6773.14653)
Supplement: Supplementary file 1 — Data S1. Items from the PCC‐CA‐36 measure arranged by subdomain with relevant statistical information. [file HESR-61-e14653-s002.docx]

Supplemental Material

|  | **Cronbach’s α** | **Range** |
| --- | --- | --- |
| **PCC-CA-36** |  |  |
| **Exchanging Information** | 0.7 | Min = 3.91  Max = 4.23 |
| How often do your doctors and other health professionals talk with you about your concerns and questions? |  |  |
| How often do your doctors and other health professionals give you helpful information, even when you don’t ask for it? |  |  |
| How often do your doctors and other health professionals make sure you have the information you need? |  |  |
| How often do your doctors and other health professionals help you understand the information you need to know? |  |  |
| How often do your doctors and other health professionals make sure your questions are answered? |  |  |
| How much do your doctors and other health professionals make you feel comfortable asking questions? |  |  |
|  |  |  |
| **Fostering Healing Relationships** | 0.72 | Min = 4.13  Max = 4.29 |
| How much can you depend on your doctors and other health professionals to give you the care you need? |  |  |
| How often do your doctors and other health professionals show they care about you? |  |  |
| How often do your doctors and other health professionals remember details about you between visits? |  |  |
| How often do your doctors and other health professionals have open and honest communication with you? |  |  |
| How much do your doctors and other health professionals seem well-informed about your type of cancer? |  |  |
| Different doctors and health professionals are often involved in a patient's care. How well do your doctors and other health professionals explain what they each do? |  |  |
| How often do your doctors and other health professionals listen carefully to what you have to say? |  |  |
|  |  |  |
| **Making Decisions** | 0.89 | Min = 4.03  Max = 4.09 |
| How often do your doctors and other health professionals involve you in making decisions about your care? |  |  |
| How well do your doctors and other health professionals explain the different choices you have? |  |  |
| How well do your doctors and other health professionals explain what they recommend? |  |  |
| How much do your doctors and other health professionals show interest in what you say about the decisions? |  |  |
| How much do your doctors and other health professionals give you information and resources to help you make decisions? |  |  |
|  |  |  |
| **Responding to Emotions** | 0.7 | Min = 4.12  Max = 4.26 |
| How often do your doctors and other health professionals give you the attention you need to your feelings and emotions? |  |  |
| How much do your doctors and other health professionals pay attention to how you are doing emotionally? |  |  |
| How much do your doctors and other health professionals show concern for your feelings, not just your illness? |  |  |
| How much do your doctors and other health professionals show concern for how your family is doing emotionally? |  |  |
| How much do your doctors and other health professionals make you feel comfortable to talk about your fears, stress, and other feelings? |  |  |
| How well do your doctors and other health professionals talk with you about how to cope with any fears, stress, and other feelings? |  |  |
|  |  |  |
| **Enabling Patient Self-Management** | 0.92 | Min = 3.76  Max = 3.97 |
| How well do your doctors and other health professionals help you understand ways you can take care of your health? |  |  |
| How much do your doctors and other health professionals talk with you about how cancer is affecting your everyday life? |  |  |
| How much do your doctors and other health professionals talk with you about ways you can manage any side effects or symptoms? |  |  |
| How much do your doctors and other health professionals talk with you about how your family can help care for you? |  |  |
| How much do your doctors and other health professionals talk with you about any concerns you have about taking care of yourself? |  |  |
| How often do your doctors and other health professionals… Make sure you understand the steps in your care? |  |  |
|  |  |  |
| **Managing Uncertainty** | 0.74 | Min = 3.81  Max = 4.17 |
| Cancer patients often face uncertainties about their cancer. For example, patients may not know what will happen, how treatment is working, and how to make sense of different information and opinions. How well do your doctors and other health professionals help you deal with the uncertainties about your cancer? |  |  |
| How much do your doctors and other health professionals help you understand if you are getting better or worse? |  |  |
| How much do your doctors and other health professionals help you understand the goal of your care? |  |  |
| How much do your doctors and other health professionals help you understand what is likely to happen with your cancer? |  |  |
| How much do your doctors and other health professionals help you understand how your symptoms may change? |  |  |
| Patients often get information from different places. How well do your doctors and other health professionals help you understand what information is most important? |  |  |

*Note: “Different response option formats were used to assess different aspects of PCC, including frequency (never, rarely, sometimes, often, always), amount (not at all, not very much, somewhat, a lot, a great deal), quality (poorly, not very well, fairly well, very well, outstanding), and presence (no, yes).”*
