# Supplementary material for: Where Can Artificial Intelligence Assist Cancer Care?: Examining Patient‐Centered Communication Dimension Effects
Source: Health Serv Res. 2025 Jun 6;61(2):e14653. doi: 10.1111/1475-6773.14653 (PMC12967909; doi:10.1111/1475-6773.14653)
Supplement: Supplementary file 2 — Appendix S1. Supporting Information. [file HESR-61-e14653-s001.docx]

Appendix A

The original model generated a perfect fit due to its being just-identified (χ^2^_(0)_ = 0, p < .01, RMSEA < .01, SRMR < .01, CFI = 1). As a result, patients’ intentions to use AI in more treatment methods were significantly associated with their overall preference of using AI over HMP (β = .48, p < .01), better sense of treatment continuity (β = .11, p < .01), and providers’ inability to give patients helpful information (β = -.11, p < .05). Other PCC dimensions, such as building rapport (β = .09, p = .14), making decisions (β = .07, p = .13), attending to emotions (β = -.01, p = .83), improving self-management (β = -.07, p = .12), and managing uncertainty (β = .01, p = .91) were not significant predictors of patients’ intentions.
